# Orthopedic Surgery Residents: How Much Do They Know About Occupational Therapy?

**DOI:** 10.7759/cureus.60263

**Published:** 2024-05-14

**Authors:** Matan S Malka, Bryanna Geiger, Kyle Angelicola-Richardson, Daniel Y Hong, Robert J Strauch

**Affiliations:** 1 Medicine, Columbia University, New York, USA; 2 Orthopaedic Surgery, Columbia University Vagelos College of Physicians and Surgeons, New York, USA

**Keywords:** survey, hand surgery, orthopedic surgery, occupational therapy, resident education

## Abstract

Background

Orthopedic hand surgeons rely on occupational therapy (OT) as a crucial part of rehabilitation following injury or surgery. Therefore, orthopedic surgeons should understand the full range of OT services. There is limited prior research on orthopedic residents’ understanding of OT in the United States. The main goal of this study is to examine how well orthopedic surgery residents grasp and perceive the role of OT, particularly in hand surgery, as integrated into their educational curriculum.

Methods

The study included all orthopedic surgery residents from a single institution (Columbia University, New York) during 2022-2023. We obtained permission from the Institutional Review Board, Department Chair, and Program Director to recruit participants. Eligible residents who agreed to participate completed questionnaires regarding their understanding of the role of OT in orthopedic surgery.

Results

Thirty subjects met the inclusion criteria. The total response rate from the residents was 14/30 (47%). The residents reported a mediocre level of familiarity with OT while also rating 4.5/5 the importance of OT in hand surgery without significant difference between postgraduate year groups. 11/14 residents reported no formal training concerning the role of OT in hand surgery. 12/14 residents reported that it would be helpful to spend time with an occupational therapist.

Conclusions

This study revealed the lack of confidence residents expressed regarding occupational therapists' roles. All residents recognized the importance of OT in hand surgery and expressed interest in shadowing occupational therapists. Residents of all levels acknowledge the crucial partnership between orthopedists and occupational therapists but lack formal education about the therapist's scope and role.

## Introduction

Occupational therapy (OT) is a healthcare profession that helps patients develop and recover the skills they need for daily living [1]. Occupational therapists are trained to assess patients' functional limitations and design personalized interventions to enhance their ability to carry out activities of daily living and instrumental activities of daily living. Through a comprehensive evaluation, therapists identify areas where patients experience difficulties, such as dressing, grooming, cooking, and household chores. By focusing on these specific challenges, OT interventions help patients regain independence and confidence in their daily routines. Occupational therapists empower patients with knowledge and skills to manage their conditions independently. By equipping patients with self-management tools, OT fosters self-reliance and promotes a sense of control over their recovery process. Occupational therapists collaborate with orthopedic surgeons, physiotherapists, nurses, and other healthcare professionals to provide comprehensive care to patients. This interdisciplinary approach ensures that patients receive well-rounded and holistic treatment, tailored to their unique needs and goals. The synergy among healthcare team members results in better outcomes and a more efficient recovery process. OT has proven beneficial in recovery from a myriad of different medical conditions as an integral part of a multidisciplinary team [2-7].

Severe hand injury and chronic pain correlate with reduced quality of life, heightened anxiety, and depression [8,9]. For this reason, hand surgeons rely on OT as an essential part of providing optimal patient care, which is not only limited to the postoperative setting. OT's focus on pain management and psychosocial support helps alleviate distress during the recovery process from injury or surgery. Furthermore, immobility and decreased use of affected limbs following orthopedic surgery can lead to secondary complications such as muscle weakness, joint contractures, and pressure sores. Occupational therapists address these issues through therapeutic exercises, adaptive equipment, and education on proper body mechanics, thus reducing the risk of complications and improving long-term outcomes. Thus, it is imperative that surgeons know the breadth of services that OT may provide in patient care. According to the ACGME program requirements for Orthopedic Surgery, adequately functioning within an inter-professional team is a CORE Competency. However, ACGME guidelines do not specify which specialties within the inter-professional team should be covered or how [10]. To our knowledge, there has been no prior literature investigating the understanding of OT among orthopedic surgeons or orthopedic residents in the United States. The main goal of this study is to examine how well orthopedic surgery residents grasp and perceive the role of OT, particularly in hand surgery, as integrated into their educational curriculum.

## Materials and methods

This is an Institutional Review Board (IRB) approved study. Inclusion criteria for this study included orthopedic surgery residents at a single institution practicing in the 2022-2023 year ranging from postgraduate year 1 (PGY-1) to PGY-5. Study members were blinded to the recruitment process, and all data and survey responses were de-identified to eliminate bias.

The first questionnaire asked for the year of residency and the second questionnaire included questions assessing the resident’s understanding of the role of OT within orthopedic surgery. The questions were intended to assess the resident’s understanding and attitude toward OT. The questionnaire was developed in collaboration with a licensed occupational hand therapist. There was a total of 11 questions; seven questions were graded on a Likert scale of 1-5 with 5 being the highest level of agreement and four questions were yes/no questions.

The survey's initial three questions assessed respondents' familiarity with OT, their understanding of occupational therapists' practice and accreditation requirements, and their comfort level in referring patients to OT. The next three questions inquired about the attitudes of the residents toward the field of OT including how important they felt OT was to the field of hand surgery, the importance of understanding OT protocols in hand surgery, and how often they have sent patients to OT. The final five questions assessed the opinions of residents about receiving education in the field of OT. Four yes/no questions were asked about OT education during residency. The last question inquired about the level of understanding of protocols used in OT in hand patients.

Recognizing the importance of addressing conflicts of interest and acknowledging residents as a vulnerable population, meticulous attention was given to these factors throughout the research process. The privacy and anonymity of each participant were upheld as paramount concerns, with stringent measures in place to safeguard their identities and responses. Faculty members involved in the study remained completely uninformed and were unable to access the data collected during the study.

Data analysis 

Statistical analysis was performed using IBM SPSS Statistics for Windows, Version 29 (Released 2023; IBM Corp., Armonk, New York, United States). Tables were produced using Microsoft Excel (Microsoft Corporation, Redmond, USA). Mean and standard deviation (SD) were calculated for continuous variables. N (%) was calculated for categorical variables. One-way analysis of variance (ANOVA) was used to compare continuous variables by group. Welch’s two-sample t-test was used to compare continuous variables. The chi-squared test was used to compare categorical variables. The significance level was set to p < 0.05. When appropriate, a 95% confidence interval is reported.

## Results

Demographics

Thirty subjects met the inclusion criteria. The total response rate was 14/30 (47%). At least 2/6 (33%) of the residents from each PGY responded to our questionnaire with the highest rate being in the PGY-4 group with a response rate of 4/6 (67%). The individual responses to each question by the respondents can be seen in Table 1. The mean scores for each response stratified by PGY are shown in Table 2.

**Table 1:** Survey Responses by Residents There are 11 total questions: Questions 1-6 and 11 are answered from 1-5 with 5 being the highest level of agreeability. Questions 7-10 are yes/no. PGY: Postgraduate year

Residency Year	PGY-1	PGY-1	PGY-1	PGY-2	PGY-2	PGY-3	PGY-3	PGY-3	PGY-4	PGY-4	PGY-4	PGY-4	PGY-5	PGY-5
How familiar do you feel you are with the field of occupational therapy and all it entails?	2	3	2	3	3	2	2	2	2	3	3	2	3	4
How well do you feel you understand the scope of practice and accreditation requirements of occupational therapists?	1	2	2	2	2	2	2	2	2	3	3	2	3	3
How comfortable are you with deciding which patients to send to occupational therapy?	1	5	3	3	4	2	3	4	4	4	4	3	4	4
How important do you feel occupational therapy is within the field of hand surgery?	3	5	4	5	5	4	4	4	4	5	5	5	5	5
In your role as a physician, how important is it for you to understand the hand therapy protocols used by the occupational therapist?	4	4	4	5	5	3	4	4	4	5	5	4	5	5
How often have you sent hand patients to occupational therapy?	5	5	4	4	4	4	4	5	5	5	4	5	5	5
Have you received any training concerning the role of occupational therapy in hand surgery?	No	No	No	No	Yes	No	No	No	No	No	No	Yes	No	Yes
Have you received any education directly from an occupational therapist during your education?	No	No	No	Yes	No	No	No	No	No	No	No	No	No	No
Do you think it would be helpful to your education to spend time with an occupational therapist?	Yes	Yes	Yes	Yes	Yes	No	Yes	No	Yes	Yes	Yes	Yes	Yes	Yes
Have you ever spent time or seen patients in an occupational therapy clinic?	No	No	No	No	No	No	No	No	Yes	No	No	No	Yes	No
How well do you understand the treatment methods and protocols involved in occupational therapy for hand patients?	2	3	2	2	2	2	3	3	2	3	3	3	3	3

**Table 2:** Mean Response by Residency Year There are 11 total questions: Questions 1-6 and 11 are answered from 1-5 with 5 being the highest level of agreeability. Questions 7-10 are yes/no. PGY: Postgraduate year

Residency Year	PGY-1	PGY-2	PGY-3	PGY-4	PGY-5	Overall	
	n=3	n=2	n=3	n=4	n=2	14	p-value
How familiar do you feel you are with the field of occupational therapy and all it entails?	2.33	3.00	2.00	2.50	3.50	2.57	0.059
How well do you feel you understand the scope of practice and accreditation requirements of occupational therapists?	1.67	2.00	2.00	2.50	3.00	2.12	<0.050
How comfortable are you with deciding which patients to send to occupational therapy?	3.00	3.50	3.00	3.75	4.00	3.43	0.780
How important do you feel occupational therapy is within the field of hand surgery?	4.00	5.00	4.00	4.75	5.00	4.50	0.144
In your role as a physician, how important is it for you to understand the hand therapy protocols used by the occupational therapist?	4.00	5.00	3.67	4.50	5.00	4.36	0.024
How often have you sent hand patients to occupational therapy?	4.67	4.00	4.33	4.75	5.00	4.57	0.294
Have you received any training concerning the role of occupational therapy in hand surgery? N who answered yes (% total)	0 (0%)	1 (50%)	0 (0%)	1 (25%)	1 (50%)	3 (21%)	0.462
Have you received any education directly from an occupational therapist during your education? N who answered yes (% total)	0 (0%)	1 (50%)	0 (0%)	0 (0%)	0 (0%)	1 (7%)	0.167
Do you think it would be helpful to your education to spend time with an occupational therapist? N who answered yes (% total)	3 (100%)	2 (100%)	1 (33%)	4 (100%)	2 (100%)	12 (86%)	0.073
Have you ever spent time or seen patients in an occupational therapy clinic? N who answered yes (% total)	0 (0%)	0 (0%)	0 (0%)	1 (25%)	1 (50%)	2 (14%)	0.435
How well do you understand the treatment methods and protocols involved in occupational therapy for hand patients?	2.33	2.00	2.67	2.75	3.00	2.57	0.294

Understanding of OT

When asked about the residents' familiarity with OT, the mean score overall was 2.57±0.65 out of 5, with the highest mean score in the PGY-5 group at 3.50±0.70 and the lowest among the PGY-3 residents at 2.00±0.00. There was a nonsignificant difference between the PGYs (p=0.059). Overall, the mean understanding of OT's practice was 2.21±0.58 with the highest being among PGY-5 at 3.00±0.00 and lowest among PGY-1 residents at 1.67±0.58. There was a significant trend in the increase in understanding as the residents advanced in their training (p<0.05). The average comfort level of the residents in referring patients to OT was 3.43±1.02 with variance in the residents’ comfort levels and no significant difference between the PGYs (p=0.78).

Attitudes about OT

There was a relative agreement among the residents about the importance of OT to the field of hand surgery in particular. The average level of importance among the residents was 4.50±0.65 and there was no significant difference among the PGY levels (p=0.14). When asked to rate how important it was to understand hand therapy protocols used by OT, there was a significant difference between the groups (p=0.024). The lowest rating was by the PGY-3 group at 3.67±0.58 and the highest by the PGY-2 and PGY-5 with a 5.00±0.00 rating in each group. There was also agreement on sending hand patients often to OT with an average score of 4.57±0.51 and no significant difference between PGY groups (p=0.29). 

Assessment of residents’ opinions about education on OT

Only 3/14 (21%) of the residents responded that they have received any training concerning the role of OT in hand surgery with no significant difference between levels of training (p=0.46). There also was no significant difference (p=0.17) when asked if the residents received any education directly from an occupational therapist with only 1/14 (7%) responding “yes.” When asked if it would be helpful for their education to spend time with an occupational therapist, 12/14 (85%) responded in the affirmative (p=0.073). There also was no significant difference (p=0.43) when the residents were asked if they spent time or saw patients in an OT clinic with only 2/14 (14%) responding that they had done so. The average level of understanding of the treatment and protocols in OT for hand patients was 2.57±0.51, indicating little understanding among all PGY levels (p=0.29).

## Discussion

We present the first study assessing orthopedic surgery residents’ knowledge and understanding of the role that occupational therapists play. In our study, it was found that orthopedic surgery residents demonstrate a limited grasp of how to optimally incorporate occupational therapists into hand surgery, despite acknowledging the significance of OT in hand surgery. Similarly, Mani and Velan found that while medical practitioners were aware of the OT component of the care team, survey respondents had an incomplete understanding of the profession [11]. A study in Nigeria assessed the awareness and knowledge of OT among medical and health science undergraduates. The study found that, like in the Indian study, the respondents had relatively little knowledge of OT [12]. Additionally, in Jordan, two studies also found limited knowledge of healthcare professionals on the role of OT [13,14]. One study assessed knowledge of OT’s role in the healthcare team in the United States, but it was done among students in nursing and physician assistant programs [15]. These respondents had a limited knowledge base concerning the spectrum of OT. 

In our study, we also found limited knowledge among orthopedic surgery residents concerning the role of OT in patient care. There was no significant difference in knowledge among PGY levels, which is likely limited by our small sample size. There was agreement on the importance of OT to hand surgery and the potential benefit of spending time with occupational therapists in residency. Most orthopedic surgery residencies in the United States today require residents to spend several months on nonorthopedic surgery rotations. While this has traditionally consisted of general surgery rotations, time spent within the OT department may instead be more beneficial for residency training. Another possibility would be to have formal didactic lectures or asynchronous module training about OT as part of the orthopedic residency curriculum. Input directly from occupational therapists about how to best address this lack of knowledge would help bridge this deficit. 

Despite a low level of confidence amongst this study’s subjects in understanding the role of occupational therapists, most reported that they frequently referred patients to OT. This disconnect underlines the challenges of multidisciplinary care and the frustrations shared by medical professionals and patients. While recognizing the need for therapy in the post-injury and postoperative period, it is equally important to direct therapy regimens to prevent complications. Whether it be selecting a passive or active protocol for flexor tendon repair or custom orthoses for nonoperative management of a humeral shaft fracture, it is paramount that orthopedic surgeons and occupational therapists communicate clearly about treatment goals and directions. Any breakdown in this process can lead to patient mistrust and poor outcomes in the long term.

The main limitation of our study is the sample size. Only 14 of the 30 residents at our institution responded to our survey, although at least two residents from each PGY level did respond. Furthermore, the study was only conducted at one institution so future studies may look at comparing OT education between institutions. We also only limited our questioning specifically to the realm of hand surgery. Future studies may assess the knowledge of OT in multiple specialties including internal medicine as well as subspecialties within orthopedics.

## Conclusions

Despite valuing OT, orthopedic surgery residents have low understanding of how to best utilize occupational therapists within hand surgery. This is largely attributable to a lack of formal education on the subject. Given the frequency of OT referrals within hand surgery and orthopedic surgery training, further studies and education on this subject are warranted.
